# Egg White-Mediated Fabrication of Mg/Al-LDH-Hard Biochar Composite for Phosphate Adsorption

**DOI:** 10.3390/molecules27248951

**Published:** 2022-12-15

**Authors:** Xiaolong Ma, Shuqi Li, He Ren, Yin Zhang, Zichuan Ma

**Affiliations:** 1 School of Environmental Science and Engineering, Hebei University of Science and Technology, Shijiazhuang 050018, China; 2 Hebei Key Laboratory of Inorganic Nano−Materials, College of Chemistry and Material Sciences, Hebei Normal University, Shijiazhuang 050024, China

**Keywords:** almond shell biochar, Mg/Al-layered double hydroxide, phosphate, adsorption

## Abstract

Phosphorus is one of the main causes of water eutrophication. Hard biochar is considered a promising phosphate adsorbent, but its application is limited by its textural properties and low adsorption capacity. Here, an adhesion approach in a mixed suspension containing egg white is proposed for preparing the hybrid material of Mg/Al-layered double hydroxide (LDH) and almond shell biochar (ASB), named L-A_E_ or L-A (with or without egg white). Several techniques, including XRD, SEM/EDS, FTIR and N_2_ adsorption/desorption, were used to characterize the structure and adsorption behavior of the modified adsorbents. The filament-like material contained nitrogen elements at a noticed level, indicating that egg white was the crosslinker that mediated the formation of the L-A_E_ hybrid material. The L-A_E_ had a higher phosphate adsorption rate with a higher equilibrium adsorption capacity than the L-A. The saturation phosphate adsorption capacity of L-A_E_ was nearly three times higher than that of L-A. Furthermore, the number of surface groups and the density of the positively charged surface sites follow the ASB < L-A < L-A_E_ order, which is consistent with their phosphate adsorption performance. The study may offer an efficient approach to improving hard biochar’s adsorption performance in wastewater treatment.

## 1. Introduction

Phosphorus is known to be an essential macronutrient in crops. Phosphorus fertilizers have been produced and applied in large numbers. At the same time, it proves that phosphorus is one of the most important causes of eutrophication in reservoirs, rivers, lakes and estuaries due to its excessive discharge [1,2,3]. It is established that once the phosphate level in aquatic environments is higher than 0.03 mg L^−1^, harmful algae bloom or red tide may occur [4,5]. Consequently, it is necessary to effectively remove phosphate in effluents from industrial fields to meet the discharging limits set by the guidelines [6,7].

Several technologies have been developed to remove phosphate from effluents, including biological treatment [8], constructed wetland [9], ion exchange [10], membrane filtration [11], chemical precipitation [12] and adsorption [13], etc. Among these methods, the adsorption approach is believed to be one of the most efficient, low-consumption, and eco-friendly techniques [14,15]. Moreover, the spent phosphate-loaded adsorbents have the potential to be used as an ancillary fertilizer as well as a soil amendment [4]. Currently, a wide variety of materials are used as adsorbents to remove phosphate from aqueous solutions, such as biomass-based carbonaceous materials [16], industrial by-products [17], natural minerals [18] and synthetic materials [19].

Considering their tremendous ecological, environmental, and agronomic benefits, biochars derived from various biomass have attracted much attention [20]. Although there are various sources, biochars derived from hardwoods and hard fruit shells (referred to hereafter as hard biochar) would be more desirable as phosphate adsorbents. Hard biochar is generally in the form of granules (columnar or flakes), making it easy to facilitate the subsequent separation and recovery with a low head pressure drop in continuous sewage treatment [21]. However, there is limited information on the adsorption of phosphate by hard biochar, mainly because of its inferior textural properties and reduced adsorption capacity of pollutants [21,22,23]. Therefore, hard biochar must be modified to enhance its phosphate adsorption activity.

The most efficient method is to purposefully incorporate some metal oxides/hydroxides into biochars, imparting a better phosphate adsorption performance to the modified biochars [24]. Layered double hydroxides (LDHs) have strong ion exchange capacities and high affinities towards anions such as phosphate and nitrate, making them an ideal option for biochar modification. Some researchers showed that the adsorption capacity of the modified biochars towards phosphate increased following the introduction of Mg/Al-LDH, Zn/Al-LDH or Mg/Fe-LDH into the biochar matrix [25,26,27]. Our previous study [21] used almond shell biochar (ASB) as representative hard biochar. A group of the Mg/Al-LDH modified ASB materials were synthesized by a two-step wet impregnation method with Mg^2+^ and Al^3+^ salts and NaOH precipitant. The results demonstrated that the Mg/Al-LDH-modified ASB could enhance the adsorption of phosphate and nitrate. Nevertheless, the Mg/Al-LDH loaded on the ASB surface had low crystallinity due to chemical co-precipitation. This may result in a decline in their textural properties and ion exchange capacities but can be avoided by alternative modification approaches. Herein, an adhesion approach in compounded suspension is proposed to prepare the hybrid material of Mg/Al-LDH and ASB. In this approach, adhesion between ASB and LDH particulates is achieved in their suspensions by using egg white as a crosslinker. This approach can maintain the crystallinity of LDH as a raw material. Hence, the main objectives of this study were: (i) preparation of the hybrid material via the adhesion approach and characterization of the structure and properties of the adsorbents; (ii) batch experimental determination of the adsorption isotherms and kinetics; and (iii) theoretical analysis of the kinetic and adsorption isotherm data. The research may offer an efficient approach to improving hard biochar’s adsorption performance in wastewater treatment.

## 2. Results

### 2.1. Comparison of Adsorption Performance of the Materials

**Figure 1:**
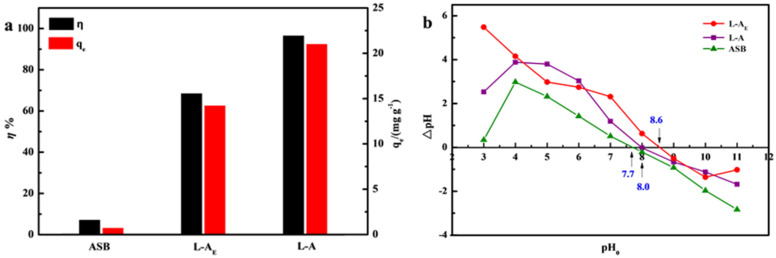
Comparison of (**a**) adsorption performance and (**b**) zero−point charge pH of the materials.

Under the condition of pH = 6.5, *m* = 2.5 g·L^−1^, *C*_0_ = 60 mg·L^−1^ and *T* = 30 °C in a total volume of 20 mL, a set of preliminary experiments was conducted to compare the adsorption ability in *η* and *q_e_* for L-A_E_, L-A and ASB (for symbols used see Section 3), and the results are shown in Figure 1a. The *η* and *q_e_* values of both L-A_E_, L-A were significantly higher than those of ASB, indicating that the introduction of Mg/Al-LDH greatly enhanced the phosphate adsorption. The *η* of L-A_E_ was 28% higher than that of L-A, and the *q_e_* value was 48% higher than that of L-A, suggesting that using egg white as a linker further enhanced the adsorption affinity of ASB/Mg/Al-LDH composite for phosphate. It is generally known that the adsorption capacity of ions (cation or anion) is positively correlated to the counter-charged surface site density of the adsorbent [28,29]. Herein, the experimentally measured pH_PZC_ values of L-A_E_, L-A and ASB were 8.6, 8.0 and 7.7 (Figure 1b), respectively, implying that all three were positively charged, but their charge density increased in the order of ASB < L-A < L-A_E_ at pH = 6.5. This explains the experimental observations presented in Figure 1a.

### 2.2. Basic Characterization of the Materials

**Figure 2:**
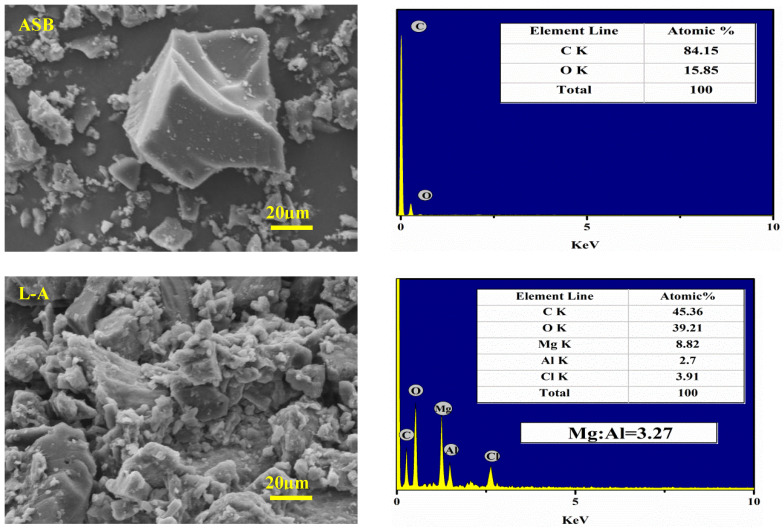

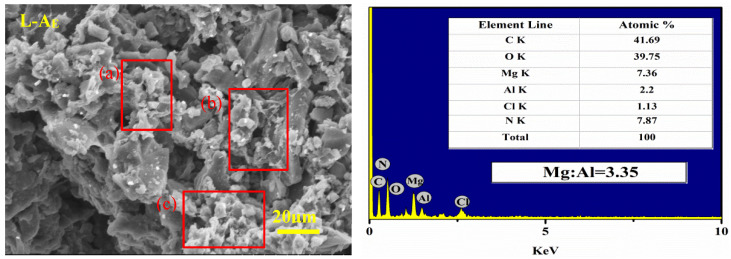
SEM/EDS analysis of the materials (red rectangles (a), (b) and (c) indicate that filament-like adhesion structures were formed in this composite).

As shown in the SEM images in Figure 2, ASB granules were poly-dispersed, irregular and polyhedral in shape. The ASB particles were formed with only carbon (84.15 at%) and a small amount of oxygen (15.85 at%), detected by EDS, which agrees with the composition of typical hard biochars [30,31].

Both L-A and L-A_E_ in Figure 2 showed the morphology of fine particles intermixed with or adhering to the ASB granules. The fine particles were identified as the Mg/Al-LDH in expected composition and crystal phases.

**Figure 3:**
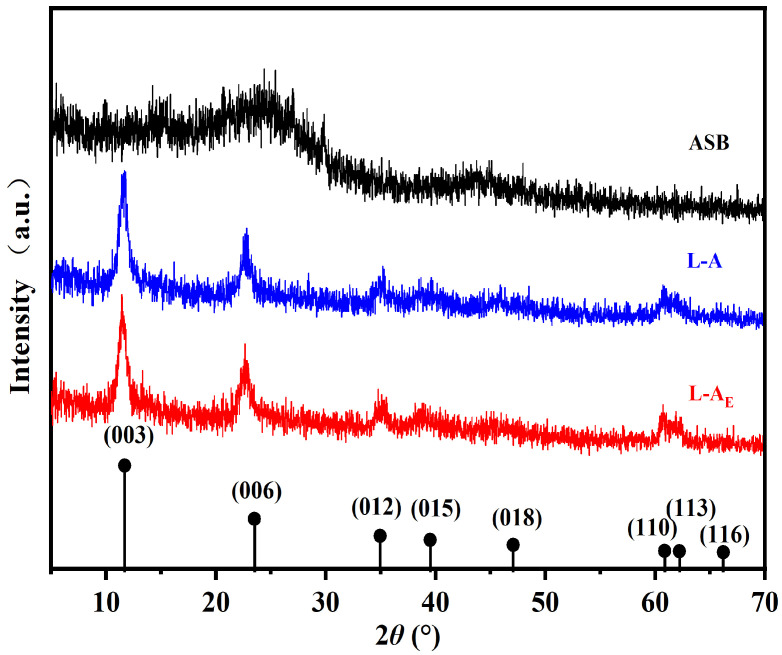
XRD patterns of the materials.

The Mg/Al atomic ratios in L-A and L-A_E_ were close to 3:1, while the XRD 2θ peaks at 11.78°, 23.52°, 34.91°, 39.48°, 47.12°, 60.86°, 62.27° and 66.26° (Figure 3) showed a typical hydrotalcite-like structure. From the SEM/EDS of L-A_E_ in Figure 2, it was also noticed that filament-like adhesion structures (in red rectangles (a), (b) and (c)) were formed in this composite. The L-A_E_ contains 7.87 at% of nitrogen, indicating that the egg white performed as a crosslinker mediated the coupling between the Mg/Al-LDH and the ASB.

**Figure 4:**
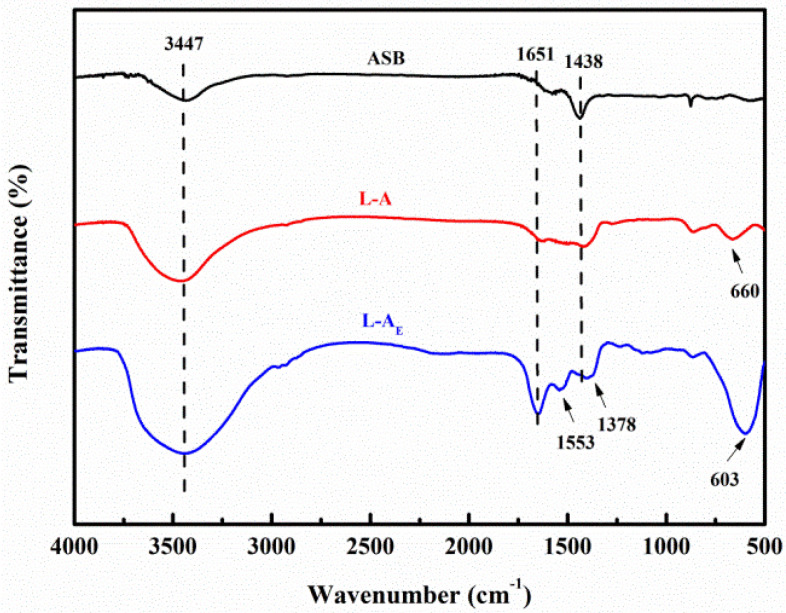
FTIR spectra of the materials.

Furthermore, with or without egg white also led to the difference in their FTIR spectra, as shown in Figure 4. Specifically, the broad band at 3453 cm^−1^ and the weak band at 1600 cm^−1^ of L-A_E_ and L-A, assigned to the O-H stretching and bending vibrations of the interlayer water molecules and hydroxyl groups in the materials structures [32], became much stronger than that of ASB. The absorption peak at 1438 cm^−1^ related to the asymmetric stretching of the CO_3_^2−^ was weakened [33] in L-A_E_ and L-A. The low-frequency peaks corresponding to the M–O and O–M–O (M = Mg, Al) vibration within 400–800 cm^−1^ (centered at 603 cm^−1^ or 660 cm^−1^ [34]) were observed in the L-A_E_ and L-A samples. It was worth noting that in the L-A_E_ FT-IR spectrum, two new peaks were detected at 1378 and 1553 cm^−1^, which could be assigned to the N–H and C–N stretching vibration from the residual egg white protein [35].

**Figure 5:**
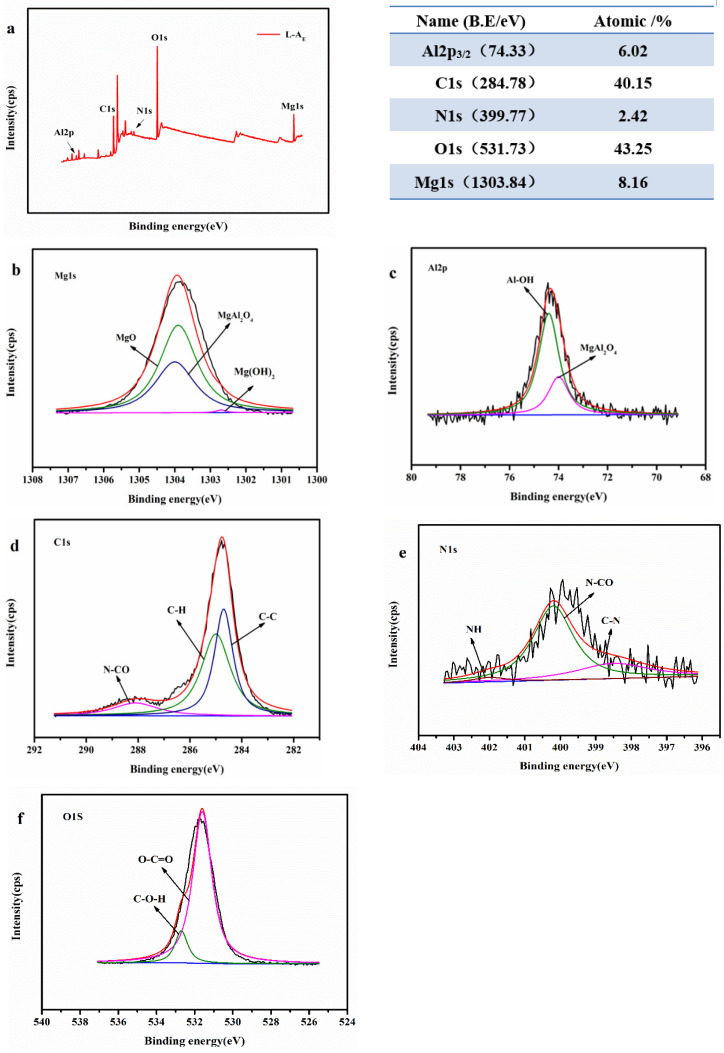
(**a**) XPS survey spectrum and high-resolution spectra of (**b**) Mg1s, (**c**) Al2p_3/2_, (**d**) C1s, (**e**) N1s and (**f**) O1s of L-A_E_.

These observations indicated that the species and number of surface groups in ASB, L-A and L-A_E_ increased in the above order, consistent with their phosphate adsorption performance. So, the surface of L-A_E_ with more abundant functional groups was further analyzed by XPS, as displayed in Figure 5. The survey spectrum in Figure 5a confirms that the L-A_E_ surface contained a significant amount of C, O, Mg, Al and N elements, which was generally consistent with its EDS result. The Mg1s spectrum (Figure 5b) was formed with three peaks at 1302.7, 1303.9 and 1304.0 eV, corresponding to the Mg(OH)_2_, MgO and Mg_2_AlO_4_ [36], respectively. The Al2p_3/2_ spectrum (Figure 5c) revealed both a Mg_2_AlO_4_ peak at 74.0 eV and an Al-OH peak at 74.4 eV [37]. The C1s spectrum in Figure 5d was deconvoluted into three peaks with the binding energies at 288.1, 285.0 and 284.7 eV, associated with the (N–CO), (C–H) and (C-C) [38], respectively. The deconvolution of the N1s spectrum in Figure 5e also resulted in three peaks at 398.5, 400.2 and 402.0 eV, attributed to the C–N, N–CO and N–H, respectively. The O1s spectrum can be deconvoluted into two peaks of O−C = O (531.6 eV) and C−O−H (532.7 eV) [39], as shown in Figure 5f. Therefore, it is concluded that the L-A_E_ contained abundant functional groups, which was beneficial to the adsorption of phosphate. However, the introduction of Mg/Al-LDH alone or in combination with egg white did not improve the textural properties of both L-A_E_ and L-A materials.

**Figure 6:**
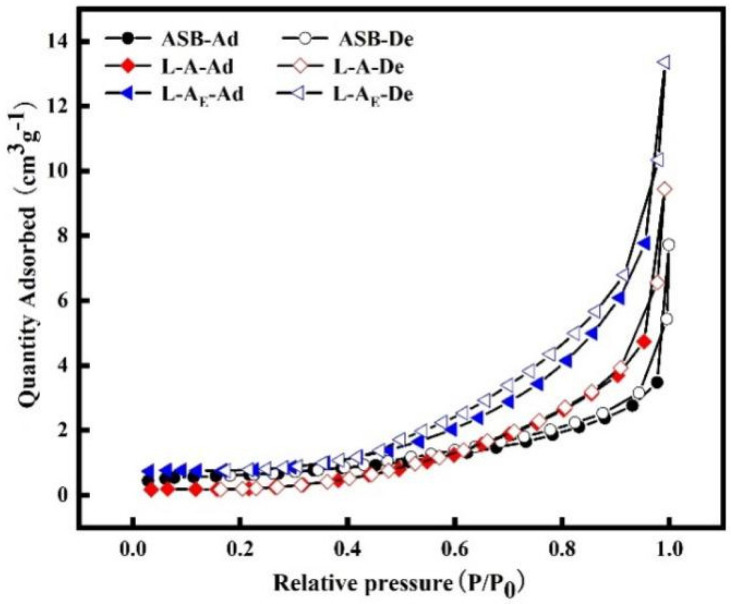
N_2_ adsorption–desorption isotherms of the materials.

From Figure 6, the N_2_ adsorption-desorption of all three materials presented the type Ⅲ isotherms indicating low nitrogen adsorption amounts due to nonporous or poor-porous structure [40].

### 2.3. Adsorption Kinetics and Isothermal Adsorption of Phosphate on L-A_E_

**Figure 7:**
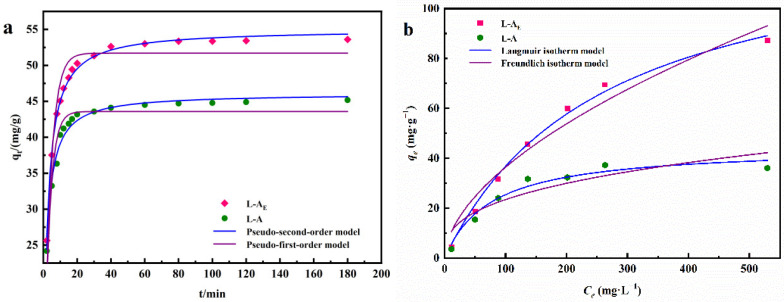
Adsorption kinetic curves (**a**) and isotherms (**b**) of phosphate on L-A_E_ and L-A.

To find the effects of the addition of egg white in phosphate adsorption, the adsorption isotherms and kinetics of both L-A_E_ and L-A materials were studied, and the results are shown in Figure 7, and the results are summarized in Table 1. Figure 7a shows that a rapid adsorption stage was observed in the first 20 min, and the adsorption equilibrium was reached at 80 min. The pseudo-second-order kinetics model gives the best fitting for phosphate adsorption on L-A_E_ and L-A with correlation coefficient (R^2^) values of 0.9956 and 0.9815, respectively. These R^2^ values were higher than those of the pseudo-first-order kinetics model (0.9158 and 0.8966). The advantage of L-A_E_ lies in its higher adsorption rate and larger equilibrium adsorption capacity than L-A. Figure 7b revealed that the equilibrium phosphate adsorption capacities of L-A_E_ and L-A were increased with the increase of initial phosphate concentration and eventually attained their respective maximum values. The Langmuir isotherm was used to analyze the adsorption capacity for L-A_E_ and L-A with R^2^ of 0.9936 and 0.9548, respectively, which are higher than the corresponding Freundlich isotherm (R^2^ of 0.9531 and 0.7967). Hence, the phosphate adsorption follows a monolayer adsorption behavior. The saturation adsorption capacity of 133.13 mg·g^−1^ from the L-A_E_ is nearly three times higher than that of L-A. The large phosphate adsorption capacity and high adsorption rate of L-A_E_ could be attributed to the introduction of egg white during the synthesis of the hybrid material, which resulted in more abundant surface elements (Mg/Al) and N/O-containing functional groups [21,41].

The high phosphate adsorption capacity of L-A_E_ is also better than some adsorbents reported in the literature (Table 2).

## 3. Experimental

### 3.1. Materials

Magnesium chloride hexahydrate (MgCl_2_·6H_2_O), aluminum chloride (AlCl_3_), potassium phosphate monobasic (KH_2_PO_4_), sodium nitrate (NaNO_3_), sodium hydroxide (NaOH) hydrochloric acid (HCl), and nitric acid (HNO_3_) were all analytical grade reagents and purchased from Beijing Chemical Reagent Company (Beijing, China). All reagents were utilized as received without further purification. Egg white was collected by separating the yolk of chicken eggs purchased from a local market (Shijiazhuang, China). Deionised (DI) water was used in all experiments. ASB was obtained from Zanhuang Carbon Material Co., Ltd. (Shijiazhuang, China). The ASB raw material was reduced to a fine powder using a multifunctional grinder before passing through a 100 mesh (0.15 mm) sieve.

### 3.2. Preparation of Adsorbents

The LDH component, with an Mg/Al atomic ratio of 3:1, was synthesized by the co-precipitation method [24], in which a 5 M NaOH solution was added dropwise under stirring to a 100 mL of 1.5 M MgCl_2_ and 0.5 M AlCl_3_ solution until the solution reached pH 9.5. The co-precipitation was continued for 2 h. After that, the precipitate was centrifugally washed to pH neutral and dried for 24 h at 105 °C. The solid sample was crushed and sieved to obtain a particle size of less than 0.15 mm.

To prepare the hybrid material between ASB and LDH with a weight ratio of 1:4 [21], 4 g of the LDH was mixed with 40 mL of an aqueous suspension containing 1 g of ASB and 5 mL of raw egg white. The mixture was kept under magnetic stirring for 2 h at room temperature before being filtered with a vacuum filter pump. The sample was dried in an oven at 105 °C for 24 h, gently crushed and sieved to select the hybrid particle size of less than 0.15 mm. Finally, the solid sample was stored in a desiccator before use. This hybrid material was labeled as L-A_E_, where the subscript “E” stressed the use of egg white as a linker. For comparison purposes, another hybrid material (L-A) of ASB and LDH was prepared following the same steps except without raw egg white.

### 3.3. Characterization of Adsorbents

Surface morphology and elemental contents of the adsorbents were analyzed using the S-4800 scanning electron microscope (SEM; Hitachi, Tokyo, Japan) equipped with an INCA 350 energy dispersive spectroscopy accessory (EDS; Oxford Instruments, Oxford, UK). The surface functional groups of the samples were determined using a Fourier transform infrared spectrometer (FTIR) of Nicolet 6700 spectrometer (Thermo Fisher, Waltham, MA, USA) with the pressed KBr tablet technique and in the range of 500–4000 cm^−1^. The crystalline phases of the materials were identified via X-ray diffraction (XRD) spectra, which were collected on a D8-Advance diffractometer (Bruker AXS, Karlsruhe, Germany) using a nickel-filtered Cu Kα radiation of 1.5406 Å at a scan speed of 1°/min in the range of 5–70°. Typical textural parameters of the as-prepared solids were determined from their N_2_ adsorption-desorption isotherms at 77 K using a surface area and porosity analyzer. Before analysis, the solid samples were degassed at 120 °C for 2 h. The specific surface area was calculated from the linear part of the Brunauer–Emmett–Teller (BET) plot (*P*/*P*_0_ = 0.05–0.35). The total pore volume was obtained from the amount adsorbed at ~0.99 of *P*/*P*_0_. The pore abundance and size distribution were assessed by Barrett–Joyner–Halenda (BJH) method for treating the desorption branch. The chemical state of surface elements in the materials was characterized by X-ray photoelectron spectroscopy (XPS), which was recorded on an ESCALAB 250Xi spectrometer with the monochromatic Al *Kα* radiation (*hν* = 1486.6 eV). The pH of the samples’ zero-point charges (pH_ZPC_) was determined using the pH Drift method [21,43].

### 3.4. Batch Adsorption Procedure

The phosphate adsorption behavior of both L-A_E_ and L-A hybrid materials was measured by batch experiments, including single point sorption, isotherm and time-dependent adsorptions. In these experiments, the dosage of adsorbents, pH and temperature in the adsorption systems were fixed at 2.5 g·L^−1^, 6.5 ± 0.1 and 30 ± 0.5 °C, respectively. During the single point sorption (the total volume is 20 mL), 60 mg·L^−1^ of initial phosphate concentration (as PO_4_^3−^) was used, and the adsorption duration was kept for 12 h; 5 mL of the suspension was extracted for phosphate analysis.

For time-dependent adsorption experiments, 400 mL of 60 mg·L^−1^ phosphate solution was transferred into a 500 mL beaker containing 2.5 g·L^−1^ of the adsorbent. The solution was sampled at the time intervals of 2, 5, 7, 10, 12, 15, 17, 20, 30, 40, 60, 80, 100, 120 and 180 min. When performing the isotherm adsorption experiments, 20 mL phosphate solutions with different initial concentrations of 10, 40, 80, 120, 160, 200, and 240 mg·L^−1^ were used to investigate the relationship between the equilibrium adsorption capacity and the phosphate concentration at constant temperature (30 ± 0.5 °C, 12 h). All the sample suspensions were separated through 0.45 μm cellulose and nylon membrane filters before the PO_4_^3−^ analysis. PO_4_^3−^ concentrations were quantified on an ion chromatograph (Qingdao Xuanhui Instruments & Equipments Co., Ltd., Qingdao, China).

The removal efficiency *η* (%), process adsorption capacity *q_t_* (mg·g^−1^) and equilibrium adsorption capacity *q_e_* (mg·g^−1^) of the adsorbent towards phosphate were calculated according to Equations (1)–(3), respectively.

(1) $$\eta =\frac{\left(C_{0}-C_{e}\right)}{C_{0}}\times 100\%$$

(2) $$q_{t}=\frac{\left(C_{0}-C_{t}\right)V}{m}$$

(3) $$q_{e}=\frac{\left(C_{0}-C_{e}\right)V}{m}$$

where *C*_0_, *C_t_* and *C_e_* are the initial, at time *t* and the equilibrium concentrations of phosphate in the solution (mg·L^−1^), respectively; *t* is the adsorption time (min); *V* is the volume of solution (L); *m* is the adsorbent mass (g).

The relationship between *q_t_* and *t* was analyzed using the pseudo-first-order and pseudo-second-order kinetic models, as shown in Equations (4) and (5). Meanwhile, the relationship between *q_e_* and *C_e_* was depicted with Langmuir adsorption isotherm (6) and Freundlich isotherm (7).

(4) $$q_{t}=q_{e}\left(1-e^{-k_{1}t}\right)$$

(5) $$q_{t}=k_{2}q_{e}^{2}t/\left(1+k_{2}q_{e}t\right)\;$$

(6) $$q_{e}=\frac{q_{m}K_{L}C_{e}}{1+K_{L}C_{e}}\;$$

(7) $$q_{e}=K_{F}C_{e}^{1/n}\;$$

where *k*_1_ (min^−1^) and *k*_2_ (g·mg^−1^·min^−1^) are the rate constants presenting the pseudo-first-order kinetic and pseudo-second-order kinetic; *q_m_* is the saturation adsorption capacity (mg·g^−1^); *K_L_* (L·mg^−1^) and *K_F_* ((mg·g^−1^) (L·mg^−1^)^1/*n*^) are the Langmuir constant and Freundlich constant, respectively; and 1/*n* is the Freundlich linearity coefficient.

## 4. Conclusions

Mg/Al double hydroxides modified ASB were prepared by the egg white adhesion approach. Using egg white as a linker mediates the formation of L-A_E_ hybrid material while maintaining the crystallinity of LDH, further enhancing the adsorption affinity for phosphate. The L-A_E_ hybrid material has a filament-like structure and more abundant surface elements (Mg/Al) and N/O-containing functional groups. The experimental adsorption data can be fitted well by the pseudo-second-order model and Langmuir isotherm, indicating that L-A_E_ has a high adsorption efficiency for phosphate and follows a monolayer adsorption behavior. The saturation adsorption capacity reached 133.13 mg·g^−1^. This work may provide an effective method of improving the adsorption performance of hard biochar from wastewater.

## Figures and Tables

**Table 1:** Kinetics and isothermal model parameters of phosphate adsorption on L-A_E_ and L-A.

Models	Parameters	Adsorbents	
		L-A_E_	L-A
Pseudo-first-order	*q_e_* (mg·g^−1^)	51.70	43.58
	*K*_1_ (min^−1^)	0.255	0.306
	R^2^	0.9158	0.8966
Pseudo-second-order	*q_e_* (mg·g^−1^)	55.01	46.09
	*K*_2_ (g·mg^−1^·min^−1^)	0.008	0.012
	R^2^	0.9956	0.9815
Langmuir	*q_m_* (mg·g^−1^)	133.13	44.71
	*K_L_* (L·mg^−1^)	0.0038	0.013
	R^2^	0.9936	0.9548
Freundlich	*K_F_* (mg·g^−1^) (L·mg^−1^)^1/*n*^	2.73	4.62
	1/*n*	0.56	0.35
	R^2^	0.9531	0.7967

**Table 2:** Phosphate adsorption capacities of different adsorbents.

Adsorbents	pH	Temperature (K)	*q_m_* (mg·g^−1^)	Ref.
Fe_3_O_4_@Zn–Al–LDH	-	298	36.9	[42]
Fe_3_O_4_@Mg–Al–LDH	-	298	31.7	[42]
Fe_3_O_4_@Ni–Al–LDH	-	298	26.5	[42]
pyromellitic acid intercalated ZnAl-LDHs	-	293	57.05	[43]
ZnFe-LDHs@Alg	-	303	94.64	[41]
20% Mg-BC	6.0	298	56.12	[28]
20% Al-BC	6.0	298	38.71	[28]
amorphous-ZrO_2_/Mg–Fe layered double hydroxide composite	7.0	290	66.08	[44]
La(OH)_3_-modified, canna-derived biochar	7.0	298	37.37	[4]
NaLa(CO_3_)_2_/Fe_3_O_4_ composites	6.8	298	77.85	[45]
MgCo_2_O_4_	5.0	303	58.69	[46]
MgO-biochar	6.0	298	18.94	[47]
L-A	6.5	303	44.71	This study
L-A_E_	6.5	303	133.13	This study

## Data Availability

Data is contained within the article.
